# Zyxin inhibits the epithelial–mesenchymal transition process in gastric cancer by upregulating SIRT1

**DOI:** 10.1002/mco2.357

**Published:** 2023-09-03

**Authors:** Jing Lou, Sha Geng, Wei He, Song‐Bai Liu, Xinghong Shi, Ying Chang, Shiyuan Han, Panting Qian, Hesham M Amin, Yao‐Hua Song, Yangxin Li, Jin Zhou

**Affiliations:** ^1^ Cyrus Tang Hematology Center Collaborative Innovation Center of Hematology Soochow University Suzhou China; ^2^ National Clinical Research Center for Hematologic Diseases the First Affiliated Hospital of Soochow University Suzhou China; ^3^ State Key Laboratory of Radiation Medicine and Protection Soochow University Suzhou China; ^4^ Suzhou Key Laboratory of Medical Biotechnology Suzhou Vocational Health College Suzhou China; ^5^ Department of Hematopathology The University of Texas MD Anderson Cancer Center Houston Texas USA; ^6^ Institute for Cardiovascular Science and Department of Cardiovascular Surgery First Affiliated Hospital and Medical College of Soochow University Collaborative Innovation Center of Hematology Soochow University Suzhou Jiangsu China; ^7^ Department of General Surgery the First Affiliated Hospital of Soochow University Suzhou China

**Keywords:** acetylation, EMT;SIRT1, gastric cancer, zyxin

## Abstract

Tumor development relies on the stemness of cancer stem cells, which is regulated by environmental cues. Previous studies have shown that zyxin can inhibit the expression of genes for embryonic stem cell status. In the present study, the expression levels of zyxin protein in cancer tissues and adjacent noncancerous tissues from 73 gastric cancer patients with different clinical stages were analyzed by Western blot. We showed that the relative expression levels of zyxin in gastric cancer tissues (cancer tissues/adjacent tissues) were significantly downregulated in advanced clinical stages. Overexpression of zyxin inhibited the stemness and epithelial–mesenchymal transition (EMT) processes in gastric cancer cells. Zyxin also inhibited the proliferation, migration, and invasion but increased the sensitivity of cancer cells to drugs. Overexpression of zyxin in MKN45 cells inhibited tumor growth in nude mice. We show that the interactions between zyxin and SIRT1 led to the upregulation of SIRT1, reduced acetylation levels of histone H3 K9 and K23, decreased transcription levels of SNAI 1/2, and inhibition of the EMT process. This study demonstrated that zyxin negatively regulates the progression of gastric cancer by inhibiting the stemness of cancer stem cells and EMT. Our findings shed new light on the pathogenesis of gastric cancer.

## Graphical abstract

The summary graph shows the role of Zyxin and histone deacetylation in regulating related EMT signal pathways. Physical interactions between zyxin and SIRT1 led to the upregulation of SIRT1, reduced acetylation levels of histone H3 K9 and K23, decreased transcription levels of *SNAI 1/2*, and inhibition of the EMT process

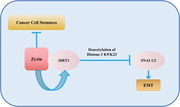

## 1 INTRODUCTION

Gastric cancer is the fifth most common cancer and the third leading cause of death worldwide.^1^ In clinical practice, gastric cancer is usually divided into stages I–IV according to the degree of infiltration of cancer cells. The 5‐year survival rate of patients with stage III gastric cancer after tumor resection is only 18%. In comparison, the 5‐year survival rates of patients with stages IA and IB disease who are actively treated with surgery are as high as 94 and 88%, respectively.^1^ Surgical resection is currently the only chance to cure gastric cancer, but even with complete resection, recurrence is common.^2^ ^[Johnston, 2019 #194]^ Systemic chemotherapy, radiotherapy, immunotherapy, and targeted therapy have all been proven to have specific effects on the clinical treatment of gastric cancer.^3^ ^[Joshi, 2021 #197]^

Zyxin is a LIM (LIN‐11, ISI‐1, MEC‐3) domain‐containing focal adhesion‐associated protein widely expressed in human tissues.^4^ It is mainly localized at the site of cell adhesion and also in the nucleus.^5^ The binding of integrins to extracellular matrix proteins induces the formation of signaling complexes at adherent plaques. Zyxin colocalizes with integrins at cell‐matrix adhesion sites and is a docking site for assembling multimeric protein complexes involved in regulating cell motility.^6^

Studies have shown that zyxin plays a tumor‐promoting role in breast, colon, and rectal cancer, melanoma, a tumor suppressor in lung and prostate cancer, and Ewing tumor cells. It was shown that myopodin‐induced suppression of prostate cancer cell migration is mediated by zyxin.^7^ There is no research report on zyxin and gastric cancer.

Cancer metastasis occurs through local invasion, intravasation to distant sites, and extravasation of circulating tumor cells.^8^ Epithelial–mesenchymal transition (EMT) is a normal developmental phenomenon that underlies embryonic morphogenesis.^9^ During EMT, cells lose epithelial characteristics and acquire mesenchymal markers, as evidenced by a decrease in E‐Cadherin and an increase in vimentin.^10^ But aberrant activation of EMT confers tumor cells with enhanced cell motility, metastatic properties, resistance to therapeutic agents, and a cancer stem cell (CSC) phenotype in epithelial‐derived carcinomas. EMT is regulated by several transcription factors, including SNAI1, SNA12, Twist 1, Twist 2, ZEB1, and ZEB2. These EMT‐promoting factors can inhibit the expression of the epithelial gene E‐Cadherin, thereby promoting EMT transformation. EMT is also regulated by SIRT1, a deacetylase. It was shown that SIRT1 inhibits renal tubular cell EMT through YY1 deacetylation in diabetic nephropathy.^11^ SIRT1 can either promote or inhibits EMT in cancer depending on cellular context and tissue origin.^12^ In the present study, we show that zyxin upregulates SIRT1, which inhibit EMT in gastric cancer.

In this study, we found that the expression of zyxin is negatively correlated with the progression of gastric cancer. We further showed that zyxin inhibits EMT by enhancing the expression of SIRT1, which in turn inhibits the transcription of *SNAI1/2* mRNA through deacetylation of histone H3 K9 and K23.

## 2 RESULTS

### 2.1 The expression of zyxin is downregulated in advanced gastric cancer

**FIGURE 1:**
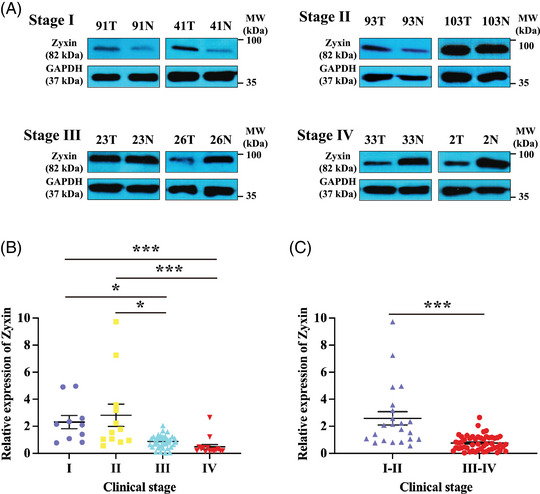
The relative expression of zyxin (cancer tissue/paracancerous tissue) was significantly decreased in patients with advanced gastric cancer. (A) Western blot analysis of relative expression of zyxin in gastric cancer patient samples at different clinical stages. *T* represents the gastric cancer area, *N* represents the adjacent noncancerous area. (B and C) Relative expression of 73 pairs of gastric cancer patient samples. Relative expression = the normalized value of cancer tissue/the normalized value of adjacent tissue. *p < 0.05, ***p < 0.001.

To determine the role of zyxin in gastric cancer, we performed a Western blot (WB) analysis of the expression of zyxin in 73 pairs of gastric cancer and adjacent tissue samples from patients with stage I‐IV of this neoplasm (Figure 1A). Our data show that the relative expression of zyxin (cancer tissue/noncancerous tissue) is significantly decreased in advanced gastric cancer tissues (Figure 1B and C), suggesting that zyxin may play a negative regulatory role in the progression of gastric cancer. We also examined zyxin expression in relation to cancer stage using the GEO and TCGA databases. As shown in Figure S1, there was no significant difference in the expression of zyxin in different stages of gastric cancer in the GEO (Figure S1A) and TCGA (Figure S1B) databases. Our results were based on the analysis of zyxin protein expression. Our WB results show that zyxin is downregulated in advanced gastric cancer tissues.

### 2.2 Clinical characteristics and prognosis of gastric cancer patients

Clinical data of 73 gastric cancer patients are shown in Table S1, including patients’ age, gender, stage, T classification, lymph nodes, and distant metastasis. In this study, correlations between clinical characteristics and zyxin protein expression in gastric cancer patients were analyzed. Our results showed that zyxin protein expression is associated with the clinical stage (*p* = 0.0007), T classification (*p* = 0.0411), lymph nodes (*p* = 0.0100), and distant metastasis (0.0012), respectively (Table 1). We also analyzed the correlation of zyxin expression with clinical features and prognosis of gastric cancer patients through the UALCAN database. We found that the expression of zyxin in gastric cancer is not related to the patient's age (Figure S2A), gender (Figure S2B), and nodal metastasis status (Figure S2C). However, compared with gastric cancer patients without TP53 mutation, the expression of zyxin is downregulated in TP53 mutated patients (Figure S2D), which further supports the inhibitory effect of zyxin in gastric cancer. In the UALCAN, TIMER, or GEPIA database, the expression level of zyxin is not closely related to the patients’ prognosis (survival time; Figure S3).

**TABLE 1:** Relationship between zyxin protein expression and clinical characteristics in the gastric cancer patients.

Characteristics	High expression of zyxin	Low expression of zyxin	*p* Value	Method
*n*	37	36		
Age, median (IQR)	68 (64, 77)	66.5 (62, 70.25)	0.0889	Wilcoxon
Gender, *n* (%)			0.9387	Chisq test
Male	26 (35.6%)	25 (34.2%)		
Female	11 (15.1%)	11 (15.1%)		
Stage, *n* (%)			0.0007	Yates' correction
I	8 (11%)	2 (2.7%)		
II	10 (13.7%)	2 (2.7%)		
III	17 (23.3%)	19 (26%)		
IV	2 (2.7%)	13 (17.8%)		
T classification, *n* (%)			0.0411	Yates' correction
T1	6 (8.2%)	0 (0%)		
T2	2 (2.7%)	2 (2.7%)		
T4	29 (39.7%)	34 (46.6%)		
Lymph nodes, *n* (%)			0.0100	Yates' correction
N0	20 (27.4%)	6 (8.2%)		
N3	8 (11%)	16 (21.9%)		
N2	6 (8.2%)	10 (13.7%)		
N1	3 (4.1%)	4 (5.5%)		
Distant metastasis, *n* (%)			0.0012	Chisq test
M0	35 (47.9%)	23 (31.5%)		
M1	2 (2.7%)	13 (17.8%)		

### 2.3 The expression of zyxin in gastric cancer cells is negatively correlated with the degree of malignancy

**FIGURE 2:**
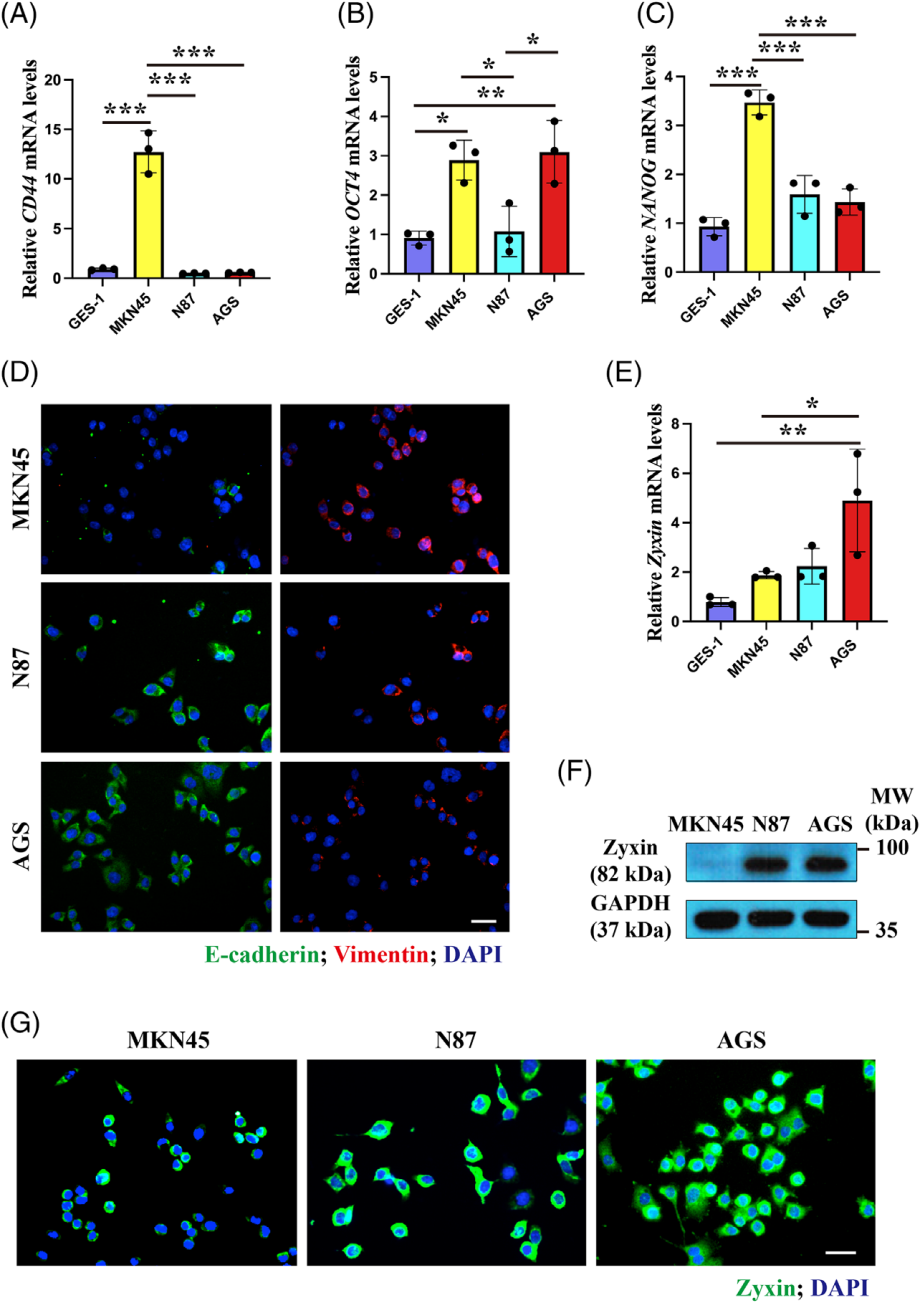
The expression of cancer stem cell marker, EMT and zyxin in gastric cancer cell lines MKN45, N87, and AGS. (A–C) RT‐qPCR analysis of *CD44*, *OCT4*, and *NANOG* mRNA. (D) Immunofluorescence staining of EMT markers E‐Cadherin and vimentin. Scale bar: 50 μm. (E) RT‐qPCR analysis of *zyxin* mRNA levels. (F) Western blot analysis of zyxin. (G) Immunofluorescence staining of zyxin. Scale bar: 50 μm. *p < 0.05, **p < 0.01, ***p < 0.001.

To determine the role of zyxin in gastric cancer, we employed three different gastric cancer cell lines: MKN45, N87, and AGS, which differ in their ability to form tumors in vivo. CSCs are a subset of cells that can initiate tumor formation. CD44 and OCT4 are the most commonly used markers for gastric CSCs.^13^, ^14^ NANOG, a cell‐fate regulatory molecule known to be important for embryonic stem cell self‐renewal, also plays an important role in tumor development. RT‐qPCR showed that the expression of *CD44*, *OCT4*, and *NANOG* mRNA was the highest in MKN45 cells (Figure 2A–C). EMT is a mechanism whereby tumors metastasize to distant sites. EMT is characterized by the downregulation of the epithelial cell marker E‐Cadherin and the upregulation of the mesenchymal cell marker vimentin. Immunofluorescence (IF) showed that E‐Cadherin expression is lowest in MKN45 and highest in AGS cells, while the expression pattern of vimentin is the opposite (Figure 2D). These results suggest that MKN45 has the highest tumorigenic potential followed by N87 and AGS.

**FIGURE 3:**
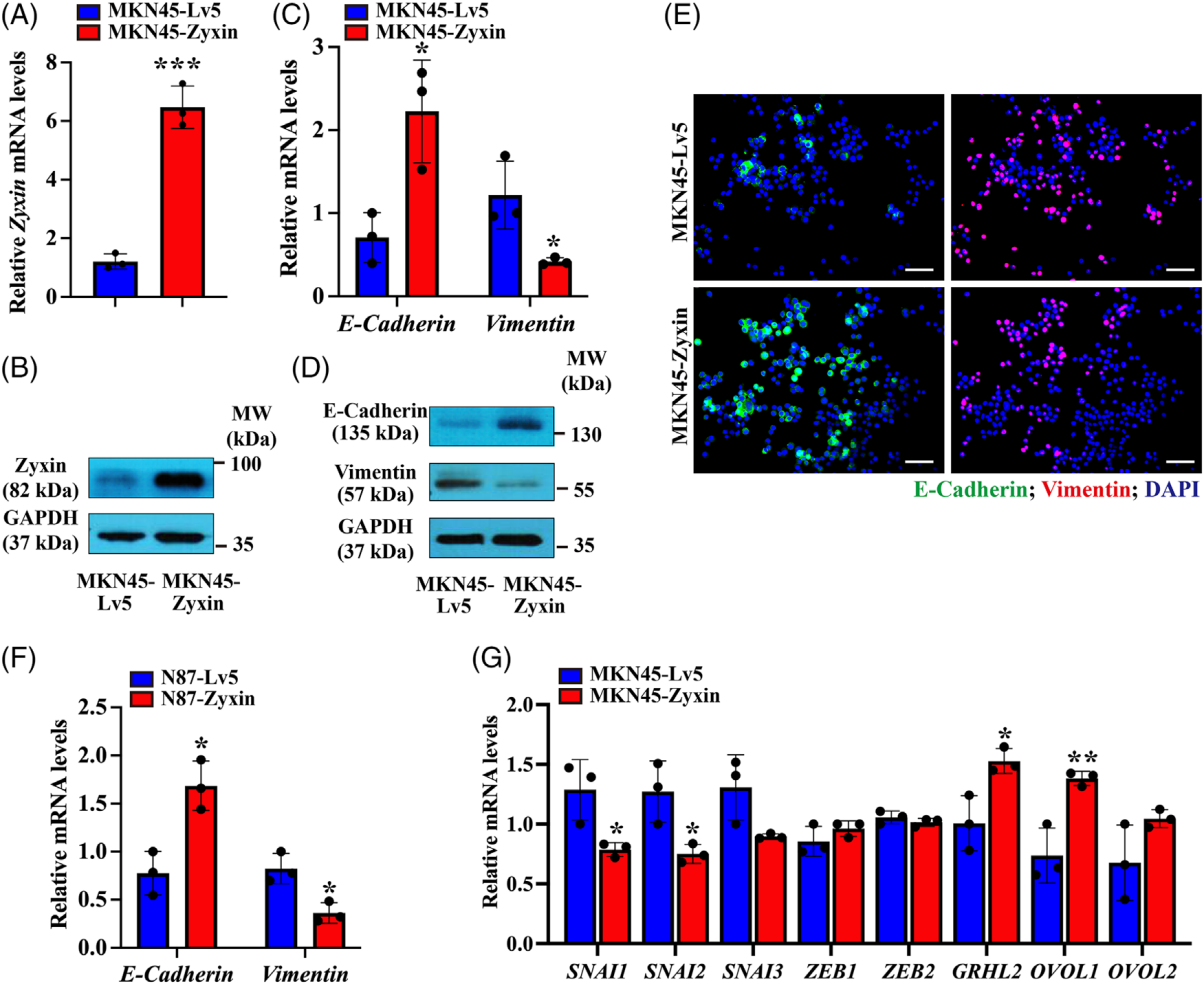
Zyxin overexpression inhibits the expression of EMT‐related markers. (A and B) RT‐qPCR and Western blot to verify the overexpression of zyxin in MKN45 cells. (C) RT‐qPCR analysis of the mRNA expression levels of *E‐Cadherin* and *vimentin*. (D) Western blot analysis of the expression of E‐Cadherin and vimentin. (E) Immunofluorescence staining of E‐Cadherin and vimentin in MKN45‐Zyxin and control MKN45‐Lv5 cells, scale bar: 100 μm. (F) RT‐qPCR analysis of *E‐Cadherin* and *vimentin* mRNA expression levels in N87‐Zyxin and control N87‐Lv5 cells. (G) RT‐qPCR analysis of EMT‐related genes in MKN45‐Zyxin and control MKN45‐Lv5 cells. Blue: MKN45‐Lv5, Red: MKN45‐Zyxin. *p < 0.05, **p < 0.01, ***p < 0.001.

However, the expression of zyxin is inversely correlated to the degree of malignancy of gastric cancer cells (Figure 2E–G), suggesting a potential inhibitory effect of zyxin on tumorigenesis. To further analyze this possibility, we overexpressed the zyxin gene in MKN45 cells (Figure 3A and B) and examined the effect of zyxin overexpression on EMT. RT‐qPCR and WB results showed that zyxin overexpression led to the upregulation of E‐Cadherin and downregulation of vimentin (Figure 3C–F). In addition, previous studies have shown that ZEB1 can induce EMT. Our WB results showed that overexpression of zyxin resulted in downregulation of ZEB1 (Figure S4). To confirm these results, we analyzed the mRNA levels of EMT‐related genes. The results showed that EMT‐promoting factors *SNAI1* and *SNAI2* were significantly downregulated, while EMT repressors *GRHL2* and *OVOL1* were significantly upregulated (Figure 3G), suggesting that zyxin negatively regulates EMT in gastric cancer cells.

### 2.4 Zyxin inhibits the expression of stem cell markers

**FIGURE 4:**
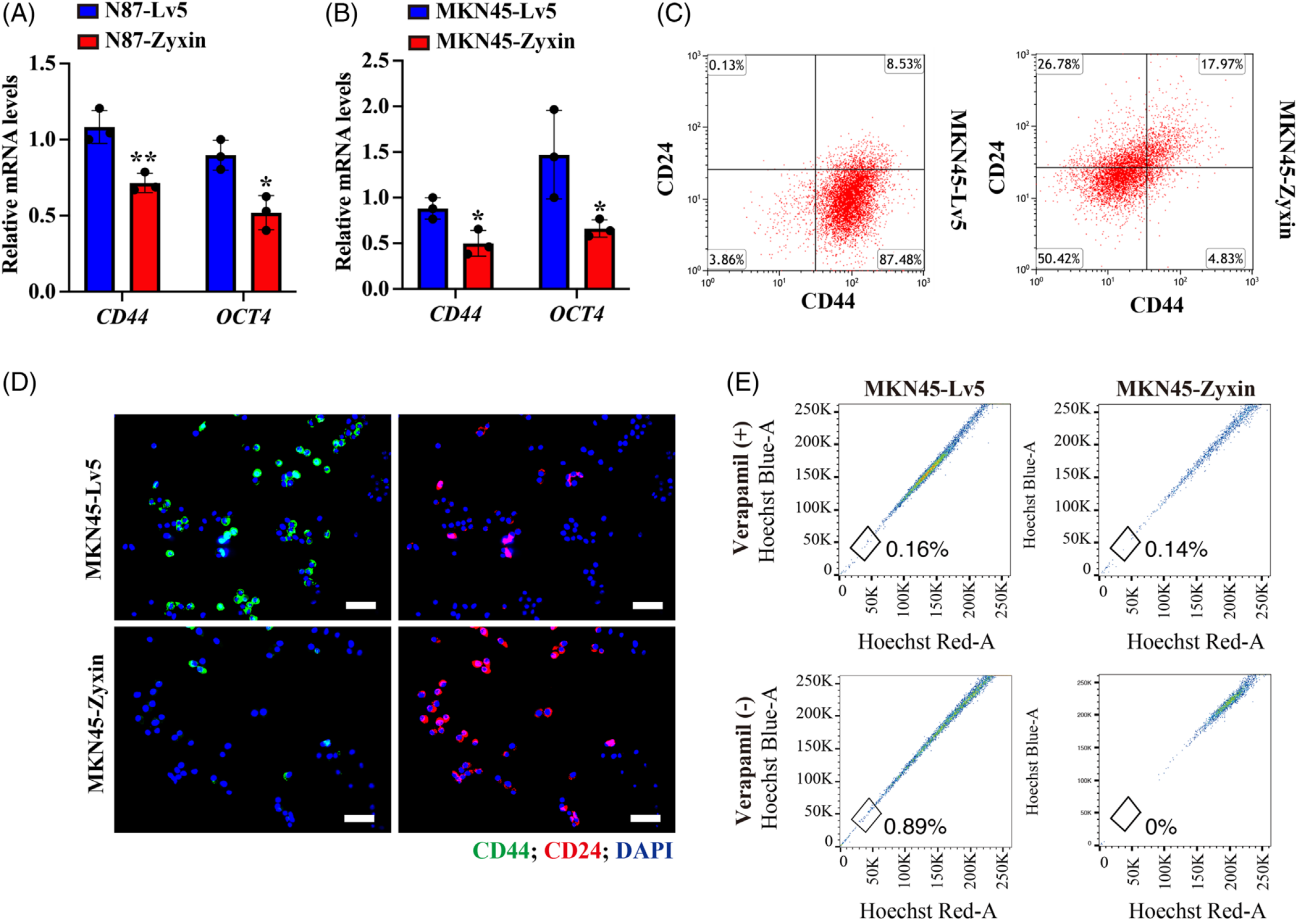
Overexpression of zyxin downregulates CD44 and upregulates CD24. (A) RT‐qPCR analysis of *CD44* and *OCT4* in N87‐Zyxin cells and control N87‐Lv5 cells. (B) RT‐qPCR analysis of *CD44* and *OCT4* in MKN45‐Zyxin cells and control MKN45‐Lv5 cells. (C) flow cytometric analysis of CD44 and CD24. (D) immunofluorescence staining of CD44 and CD24 in MKN45‐Zyxin and control MKN45‐Lv5 cells. Scale bar: 100 μm. (E) The Hoechst staining and flow cytometry were used to analyze the side population proportion in MKN45‐Lv5 and MKN45‐Zyxin cells. **p < 0.01.

To explore the effect of zyxin on tumor stemness, we overexpressed zyxin in N87 and MKN45 cells and analyzed expression levels of CD44 and OCT4. RT‐qPCR showed that the levels of *CD44* and *OCT4* mRNA are significantly decreased when zyxin is overexpressed (Figure 4A and B). The gastric CSCs are characterized by increased expression of *CD44* and decreased expression of *CD24*. Flow cytometry (FC) revealed that the overexpression of zyxin leads to decreased CD44^+^ cells and increased CD24^+^ cells (Figure 4C). These findings were confirmed by IF staining for CD44 and CD24 (Figure 4D). The Hoechst staining and FC were employed to analyze the side population in MKN45‐Lv5 or MKN45‐Zyxin cells. The results showed that zyxin overexpression reduced the SP proportion in MKN45 cells as compared with the control groups (Figure 4E). These results indicate that zyxin inhibited the stemness of gastric CSCs.

### 2.5 Zyxin inhibits the migration and invasion of gastric cancer cells

**FIGURE 5:**
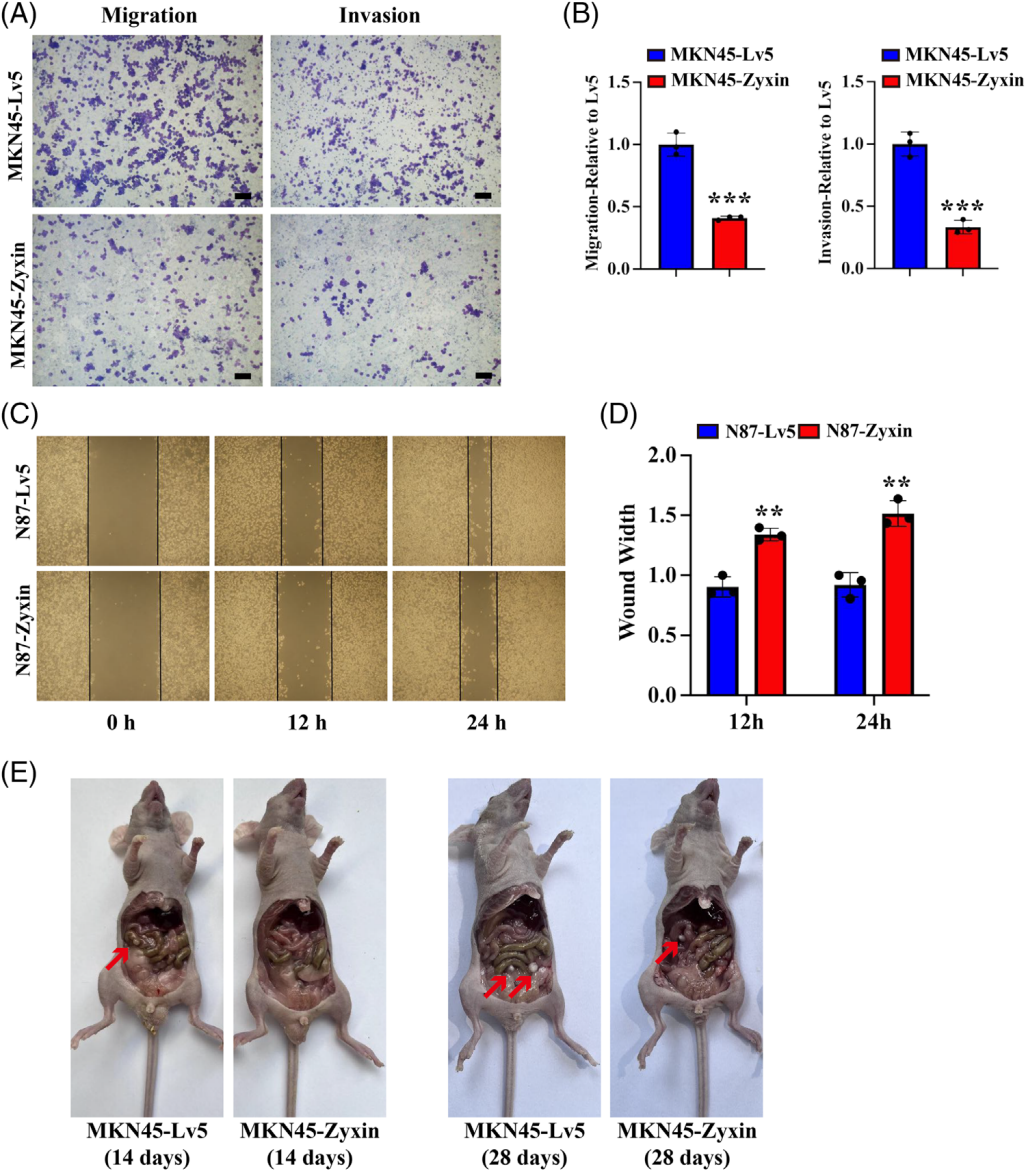
Zyxin overexpression inhibits the migration and invasion of gastric cancer cells. (A and B) Transwell assay to assess the migration and invasion ability of MKN45‐Zyxin and control MKN45‐Lv5 cells, scale bar: 50 μm. (C) The migration ability of N87‐Zyxin and control N87‐Lv5 cells was analyzed by wound‐healing assay. (D) The wound width was quantified by Image J software to show the migration ability of cells. (E) Peritoneal tumor development at varying time points in nude mice following injection with MKN45‐Lv5 or MKN45‐Zyxin.**p < 0.01, ***p < 0.001.

The migration and invasion of cancer cells were tested using a transwell assay. In the transwell assay, the number of cells passing through the chamber represents migration, whereas the number of cells passing through the Matrigel‐coated chamber represents invasion. The results showed that the migration and invasion ability is reduced by zyxin overexpression in MKN45 cells (Figure 5A and B). The same result was observed in N87 cells (Figure S5A and B). To verify the results from the migration assay, we performed a wound‐healing assay. The results showed that the migration ability is significantly decreased by zyxin overexpression (Figure 5C and D). The time to peritoneal tumor occurrence was different in MKN45‐Lv5 or MKN 45‐zyxin cells, with tumors occurring at ∼2 weeks in the MKN45‐Lv5 cells and ∼28 days in the MKN45‐Zyxin cells. This experiment demonstrated that zyxin inhibits metastasis of gastric cancer (Figure 5E).

### 2.6 Zyxin inhibits proliferation, anchorage‐independent growth, and drug resistance of gastric cancer cells

**FIGURE 6:**
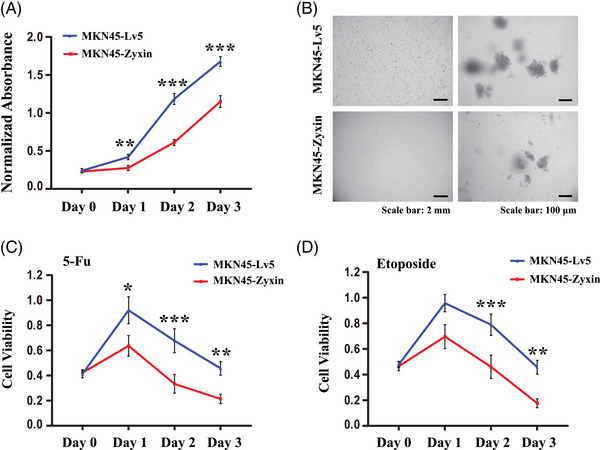
Zyxin overexpression inhibits the growth and drug resistance of gastric cancer cells. (A) The ability of MKN45‐Zyxin and control MKN45‐Lv5 cells to proliferate in vitro was analyzed by CCK8 reagent. (B) The anchorage‐independent growth of MKN45‐Zyxin and control MKN45‐Lv5 cells was detected by using a soft agar colony formation assay. The scale bar in the left panel represents 2 mm, and the scale bar in the right panel represents 100 μm. (C) The sensitivity of MKN45‐Zyxin and control MKN45‐Lv5 cells to the chemotherapeutic drugs 5‐FU (4 mM) and (D) etoposide (80 μM) was analyzed by CCK8 reagent. *p < 0.05, **p < 0.01, ***p < 0.001.

The development of gastric cancer largely depends on the growth potential of gastric cancer cells. We evaluated the proliferation of MKN45‐Zyxin/N87‐Zyxin and the control MKN45‐Lv5/N87‐Lv5 cells in vitro using the CCK8 cell proliferation assay. The results showed that zyxin overexpression significantly decreased cell proliferation (Figures 6A and S6A). Anchorage‐independent growth is one of the hallmarks of CSCs, which can be tested using the soft agar assay. The results showed that overexpression of zyxin resulted in a significant decrease in the number and size of colonies formed by MKN45 (Figure 6B) or N87 (Figure S6B) cells in soft agar.

The resistance of tumors to chemotherapy is mainly due to the presence of CSCs. 5‐Fluorouracil (5‐FU) and etoposide chemotherapeutics are commonly used to treat gastric cancer. The results showed that zyxin overexpression increased the sensitivity of MKN45 (Figure 6C and D) or N87 (Figure S6C and D) cells to the two drugs.

### 2.7 Zyxin inhibits gastric cancer tumor growth in nude mice

**FIGURE 7:**
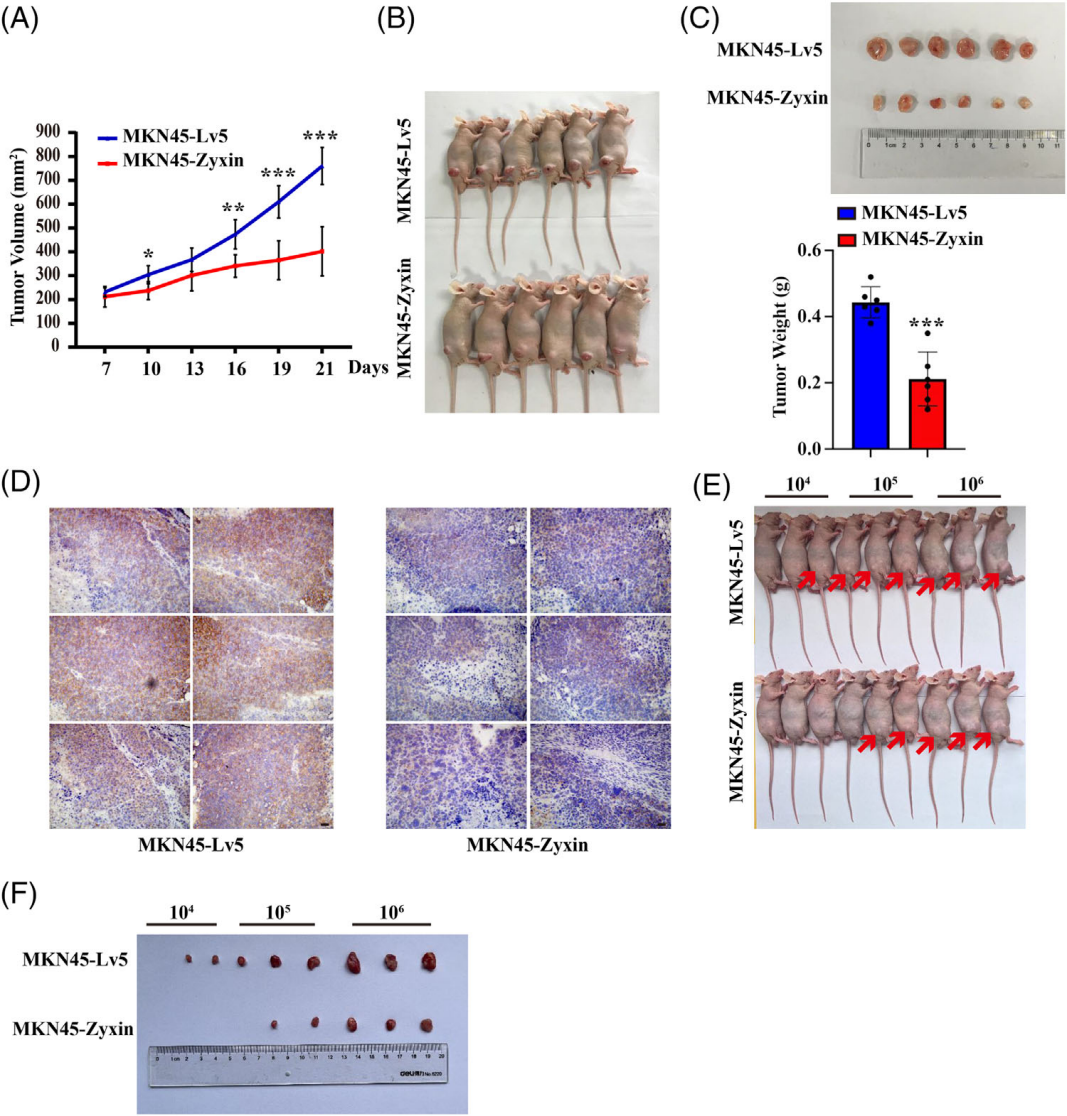
Zyxin overexpression inhibits tumor growth in nude mouse xenograft model. (A) MKN45‐Zyxin and control MKN45‐Lv5 cells were injected subcutaneously in nude mice. The tumor volumes were measured on day 21. (B and C) On day 21, the tumors were taken out, excised, and weighed. *N* = 6/group. (D) Immunohistochemical staining was performed using an anti‐CD44 antibody. Six individual panels represent tumors from six different nude mice in the same group. Scale bar: 20 μm. (E and F) The image of tumor cell gradient dilution subcutaneous inoculation tumorigenesis experiment. *p < 0.05, **p < 0.01, ***p < 0.001.

We used a mouse xenograft model to assess the impact of zyxin overexpression on tumor growth in vivo. Ten million MKN45‐Zyxin or control MKN45‐Lv5 cells were mixed with Matrigel, and injected subcutaneously in the back of adult male nude mice. Tumor volumes were measured starting on day 7 postinjection. On day 21, tumors were excised and weighed. The results showed that the tumors from the MKN45‐Zyxin group were significantly smaller than the control MKN45‐Lv5 group (Figure 7A–C). Immunohistochemical staining showed decreased CD44 expression in the tumors after zyxin overexpression (Figure 7D). When 10^4^, 10^5^, and 10^6^ MKN45‐Lv5 cells were inoculated subcutaneously into nude mice, their tumor formation rates were 33.3, 100.0, and 100.0%, respectively, while the tumor formation rates of MKN45‐Zyxin cells were 0, 66.7, and 100.0% (Figure 7E and F).

### 2.8 Zyxin suppresses EMT by upregulating SIRT1 and decreasing histone acetylation

**FIGURE 8:**
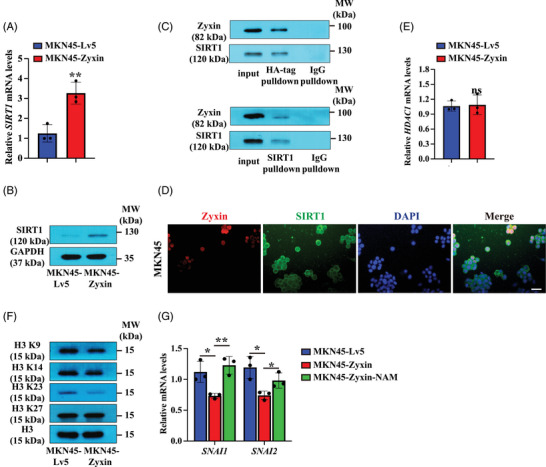
Zyxin inhibits EMT by upregulating SIRT1. (A and B) RT‐qPCR and Western blot analysis of the expression of SIRT1 in MKN45‐Zyxin and control MKN45‐Lv5 cells; (C) Western blot analysis of zyxin and SIRT1 in protein products immunoprecipitated by antibodies against HA‐tag and SIRT1 or control IgG. Left panel: proteins were pulled down by HA antibody, then analyzed by Western blot using antibodies against zyxin or SIRT1; right panel: proteins were pulled down by SIRT1 antibody, then analyzed by Western blot using antibodies against zyxin or SIRT1. (D) Immunofluorescence staining of zyxin and SIRT1 in MKN45 cells. Scale bar: 50 μm. (E) RT‐qPCR analysis of the relative expression of *HDAC1* mRNA in MKN45‐Zyxin and control MKN45‐Lv5 cells. (F) Western blot analysis of acetylation levels of H3 K9, K14, K23, and K27 sites in MKN45‐Zyxin and control MKN45‐Lv5 cells. (G) RT‐qPCR detection of the relative mRNA expression levels of *SNAI1* and *SNAI2* in MKN45‐Lv5, MKN45‐Zyxin, and MKN45‐Zyxin cells treated with NAM. *p < 0.05, **p < 0.01, ***p < 0.001.

Zyxin has been shown to be an interacting partner of SIRT1 (sirtuin 1).^15^ We examined the relative expression levels of SIRT1 in MKN45‐Zyxin and the control MKN45‐Lv5 cells, and the results showed that overexpression of zyxin in MKN45 cells upregulated SIRT1 (Figure 8A and B). To further confirm the interaction between SIRT1 and zyxin, we performed co‐immunoprecipitation (Co‐IP) experiments using MKN45 cells overexpressing HA‐zyxin and antibodies against HA‐tag (zyxin overexpression plasmid fusion tag), SIRT1, and normal IgG as control. The results showed that zyxin and SIRT1 are detected in the protein product precipitated by the HA and SIRT1 antibodies, suggesting a physical interaction between zyxin and SIRT1 in MKN45 cells (Figure 8C). In addition, our IF experiments indicate that the zyxin colocalizes with SIRT1‐positive in the MKN45 cells (Figure 8D).

Since SIRT1 is a nicotinamide adenosine dinucleotide‐dependent deacetylase that regulates gene expression by histone deacetylation,^16^ we investigated the potential involvement of another class of deacetylase—HDAC1. Both SIRT1 and HDAC1 have been implicated in cancer development.^17^ It was shown that SIRT1 inhibits EMT in cancer metastasis.^18^ RT‐qPCR showed that zyxin overexpression had no effect on the relative expression level of *HDAC1* mRNA (Figure 8E).

Our results show that the acetylation levels of histone H3 K9 and K23 sites were significantly reduced when zyxin was overexpressed (Figure 8F), suggesting that SIRT1 may inhibit EMT by deacetylating histone H3 K9 and K23 sites.

Overexpression of zyxin resulted in a reduction of *SNAI1* and *SNAI2* expression, which was rescued by NAM (Figure 8G). These results suggest that zyxin inhibits EMT by downregulating the acetylation of histones H3 K9 and K23 sites through the upregulation of SIRT1.

## 3 DISCUSSION

Zyxin is an adhesion plaque protein that regulates cell adhesion and motility and also transmits signals from the extracellular environment to the nucleus to regulate important processes such as cell proliferation. Zyxin also plays an important role in the initiation and development of cancer.^19^, ^20^ The results of this study show that zyxin can inhibit the stemness and EMT of gastric cancer cells.

Our results in this paper show that zyxin overexpression leads to increased expression of *SIRT1* mRNA and protein, whereas the relative expression of *HDAC1* mRNA remains unchanged. Through Co‐IP experiments, we found that zyxin and SIRT1 can physically bind in gastric cancer cells MKN45, which indicates that zyxin may inhibit the initiation and development of gastric cancer through SIRT1. We further showed that the overexpression of zyxin induces a reduction of SNA11 and SNAI2 expression, which is rescued by NAM. Our results are consistent with previous reports that SIRT1 suppresses EMT in cancer by deacetylating Smad4 and inhibiting TGF‐β signaling in breast cancer and oral squamous cell carcinoma.^18^

The specific mechanisms by which zyxin regulates the expression of gastric cancer cell stemness marker CD44 remain unknown. It was shown that CD44 expression is regulated by TCF‐4, β‐catenin, Ras, ERK1/2, NF‐kB, Akt, TGF‐β, and Nanog signaling pathways.^21^ In addition, CD44 has two isoforms (CD44s and CD44v) resulting from alternative splicing. It was shown that both CD44s and CD44v are involved in tumor initiation and EMT in breast cancer.^22^, ^23^, ^24^ Depletion of CD44s reduces tumor cell invasion and metastasis.^25^ The conversion of CD44s to CD44v promotes tumorigenesis.^26^ High CD44 expression was observed in gastric cancer, and its expression correlates with immune cell infiltration.^27^ Zyxin is also involved in immune cell infiltration in ulcerative colitis.^28^ Additional studies are needed to further explore the impact of CD44 isoforms on the development of gastric cancer.

OCT‐4 is a transcription factor protein encoded by the *POU5F1* gene, belonging to the POU (Pit‐Oct‐Unc) family.^29^ It is a marker for embryonic stem cells and maintains pluripotency by promoting self‐renewal.^30^, ^31^ OCT‐4 is one of the factors required for generating induced pluripotent stem cells.

Both embryonic stem cells and CSCs have the ability of unlimited growth, and it is not surprising that OCT4 can be detected in tumors.^32^ OCT‐4 is a tumor suppressor in breast cancer but an oncogene in kidney and ovarian cancers.^33^ OCT‐4 has eight pseudogenes and some of which have been implicated in cancer development. POU5F1P3 and POU5F1P4 are oncogenes in kidney and colorectal cancer, respectively.^33^ A recent study showed that the POU5F1B promotes an aggressive phenotype of gastric cancer.^34^

Our data demonstrated an inverse relationship between Oct4 and zyxin, which is in line with a recent report showing that the downregulation of zyxin in embryonic stem cells enhances the expression of OCT4.^35^

There are limitations of this study too. In the mechanism experiment, we did not provide a specific mechanism study on how zyxin regulates SIRT1 expression. Previous literature indicates that zyxin is a novel interacting partner for SIRT1. SIRT1 and zyxin transcript were both preferentially expressed in the developing mouse brain. Zyxin accumulates in the nucleus, where it is colocalized with SIRT1 after treatment with leptomycin B in COS‐7 cells. Furthermore, SIRT1 deacetylates zyxin, suggesting SIRT1 could interact with nuclear‐accumulated zyxin and modulate its function through deacetylation. However, the specific mechanism study on how zyxin regulates SIRT1 expression is unknown, it is also one of the limitations of this study. We can explore this problem through the RNA‐Seq method in the future.

Microsequence analysis identified cell cycle and apoptosis regulator protein‐1 (CARP‐1) as a zyxin‐binding partner.^36^ CARP‐1 was required for retinoids and doxorubicin‐induced tumor cell death.^37^ Cells harboring a homozygous disruption of the zyxin gene are resistant to UV‐induced apoptosis.^36^ However, zyxin lacking the CARP‐1 binding region shows reduced proapoptotic activity in response to UV‐C irradiation, suggesting that CARP‐1 and zyxin cooperate to promote apoptosis.^36^ CARP‐1 was downregulated in gastric cancer tissue compared to the normal gastric tissue.^38^ Overexpressing CARP‐1 suppressed the malignancy of gastric carcinoma BGC‐823 cell line by inducing apoptosis.^38^ Future studies are required to explore whether CARP‐1 is essential in mediating the tumor‐suppressing effect in gastric cancer.

The Hedgehog and Notch signaling pathway and the Farnesoid X receptor (FXR) are involved in tumor initiation and CSCs, as discussed by Xia and Girisa et al.^39^, ^40^ Future studies should explore whether zyxin could regulate the Hedgehog/Notch and FXR signaling pathway.

In conclusion, this study demonstrated a negative relationship between zyxin expression and the development of gastric cancer. Zyxin reduces gastric cancer cell stemness and EMT by inhibiting the acetylation level of histone H3 K9 and K23, resulting in the downregulation of SNAI1/2. These findings shed new light on the pathogenesis of the development of gastric cancer. Therapeutic strategies targeting zyxin should be exploited to eradicate CSCs for cancer treatment.

## 4 MATERIALS AND METHODS

### 4.1 Study design and participants

To investigate the role of zyxin in gastric cancer, we collected samples of gastric cancer patients at different stages from the First Affiliated Hospital of Suzhou University in Jiangsu Province, China. Gastric cancer tissues and adjacent tissues were collected from 73 gastric cancer patients diagnosed by histopathology and underwent radical gastric cancer resection in the hospital from August 2019 to May 2020. The patients did not receive chemotherapy or radiotherapy before surgery. The gastric cancers were staged according to the 2010 WHO Digestive System Tumor Classification Standard. All samples were immediately frozen in liquid nitrogen and stored until analysis.

### 4.2 Animals

BALB/c‐Nude mice (SPF grade, 6−8 weeks old, male) were purchased from Jiangsu Jicui Yaokang Biological Co., Ltd. All mice used in this study were males from 8 weeks and maintained in a Special Pathogen Free animal facility at Soochow University. Mice were kept in ventilated cages with filter tops in groups of six, The mice have access to drinking water and standard chow ad libitum. The temperature in animal facilities were kept at 22–26°C, and the humidity was maintained at 55 ± 10%. All animal experiments comply with the ARRIVE guidelines (NIH Publications No. 8023, revised 1978) and were approved by the Institutional Laboratory Animal Care and Use Committee of Soochow University.

### 4.3 Cell lines

Human gastric cancer cell line MKN45 purchased from Beijing Institute of Cell Biology, Chinese Academy of Sciences was grown in 1640 medium with 10% FBS. Human gastric cancer cell lines N87 and AGS purchased from Shanghai Institute of Cell Biology, Chinese Academy of Sciences were cultured in RPMI and F‐12K medium (65%F‐12K, 25% 1640, 10% FBS), respectively. Human normal gastric epithelial cell line GES‐1 (Shanghai Fuheng Biotechnology Co., Ltd.) and human renal epithelial cells 293T (Shanghai Institute of Cell Biology, Chinese Academy of Sciences) was cultured in DMEM medium with 10% FBS.

### 4.4 Antibodies

Zyxin antibody was from Santa Cruz (sc‐293448) at 1:250 dilution for WB, 1:100 dilution for IF). The following antibodies were from Cell Signaling Technology: ZEB1 (3396S, 1:1000 dilution for WB), GAPDH (2118S, 1:5000 dilution for WB), E‐Cadherin (14472S, 1:1000 dilution for WB, 1:100 dilution for IF), vimentin (5741S, 1:1000 dilution for WB, 1:100 dilution for IF), CD44 (3570S, 1:300 dilution for immunohistochemistry [IHC]), 1:400 dilution for IF), SIRT1 (8469S, 1:1000 dilution for WB, 1:100 dilution for IP), 1:100 dilution for IF), HA‐Tag (3724S, 1:50 dilution for IP), anti‐mouse IgG (HRP‐linked, 7076S, 1:5000 dilution for WB), anti‐rabbit IgG (HRP‐linked, 7074S, 1:5000 dilution for WB). CD44‐PE (550989, 1:50 dilution for FCM) and CD24‐PerCP‐Cy5.5 (561647, 1:50 dilution for FCM) were from BD; CD24 (ab202073, 1:100 dilution for IF), H3 K9 (ab4441, 1:10000 dilution for WB), H3 K14 (ab82501, 1 mg/mL for WB), H3 K23 antibody (ab177275, 1:1000 dilution for WB), H3 K27 Antibody (ab4729, 1 μg/mL for WB), and H3 (ab47915, 1:1000 for WB) were from Abcam. Alexa Fluor 488 goat anti‐mouse IgG1 (A21121, 1:500 dilution for IF), Alexa Fluor 488 goat anti‐mouse IgG(H+L) (A11001, 1:500 dilution for IF), Alexa Fluor 568 goat anti‐rabbit IgG(H+L) (A11010, 1:500 dilution for IF), and Alexa Fluor 568 goat anti‐mouse IgG2a (A21134, 1:500 dilution for IF) were from Thermo Fisher Scientific.

### 4.5 Western blot

Cell or tissue samples were solubilized in lysis buffer (Cell Signaling Technology; 46232S), then centrifuged to remove the insoluble particles. After separation by SDS‐PAGE, the proteins were transferred onto a PVDF membrane. After blocking with 5% nonfat milk, the membranes were incubated with primary and secondary antibodies. The bands were visualized with ECL chemiluminescence reagent (PerkinElmer; NEL120001EA).

### 4.6 IF staining

The cells were fixed with immunostaining fixative solution (Beyotime; P0098) and permeabilized with Triton X‐100 solution (Sigma–Aldrich; 9002‐93‐1). After blocking with the immunostaining blocking solution (Beyotime; P0102), the cells were incubated with the primary and secondary antibodies, and the nuclei were stained with DAPI (Sigma–Aldrich; D8417) as previously described.^41^

### 4.7 RT‐qPCR

RNA was extracted using the Trizol solution (Invitrogen; 15596026). RT‐qPCR primers were designed on the Primer Bank website (https://pga.mgh.harvard.edu/primerbank/) and on the NCBI‐Primer BLAST website (https://www.ncbi.nlm.nih.gov/tools/primer‐blast) for verification. The primers were synthesized by Hongxun Biological Co., Ltd. The Ct value was detected by real‐time fluorescence quantitative PCR instrument Quant Studio 6, and the relative expression level of mRNA was calculated according to the 2^−ΔΔCt^ method. The RT‐qPCR primer sequences are shown in Table S2.

### 4.8 Construction of zyxin overexpressing cells

Zyxin cDNA was amplified with primers containing the *Sph* I and *Bam*H I restriction site sequence and HA tag by using the KOD enzyme reaction system (Toyobo; KOD201). The product cloned into Lv5 plasmid (GenePharma). The gastric cancer cells were transduced with lentivirus packaged in 293T cells and screened with puromycin as described previously.^42^ The CDS sequences of zyxin and the sequences of zyxin overexpression PCR primers are shown in Table S3.

### 4.9 FC analysis

We collected 1 × 10^7^ gastric cancer cells for FC analysis. After incubating with the primary antibodies, the cells were washed and filtered through a 70 μm filter and analyzed by flow cytometer.

### 4.10 Side population

The side population from the MKN45‐Lv5 or MKN45‐Zyxin cells was analyzed by labeling the cells with Hoechst 33342 dye (Sigma; B2261) as described previously with modifications.^43^ The cells resuspended in RPMI‐1640 medium (10% FBS) was incubated with the Hoechst 33342 dye with or without verapamil (100 μmol/L) at 37°C for 90 min.

### 4.11 Scratch test

The cells were cultured in a six‐well plate. A straight line was drawn at the bottom of the plate using a 100 μL pipette tip. The images were taken at 0, 12, and 24 h using a biological image navigator.

### 4.12 Migration and invasion assays

The migration and invasion assays were performed using transwell chambers as described previously.^41^

### 4.13 Cell proliferation assay

Cell proliferation was determined using the CCK8 reagent as we have described previously.^41^ Drug sensitivity assay was performed on 96‐well plates. MKN45‐Lv5 and MKN45‐Zyxin cells were treated with 5‐FU (4 mM) and etoposide (80 μM) before CCK8 reagent was added.

### 4.14 Soft agar colony formation assay

Soft agar assay was performed in a petri dish containing two layers of agarose as we have described previously.^41^

### 4.15 Nude mice xenograft tumors experiment

Gastric cancer cells (1 × 10^7^) mixed with 100 μL Matrigel were injected subcutaneous on the back of the nude mice as described previously.^41^ On day 7, tumor was measured (tumor volume *V* = *π*/6 × length × width^2^), and the tumor was taken out and photographed, weighed, and measured on day 21.

### 4.16 Analysis of tumorigenic ability in vivo

Different amount of MKN45‐Lv5 or MKN45‐Zyxin cells (10^4^, 10^5^, and 10^6^) were mixed with Matrigel, and injected subcutaneously into nude mice. The tumor formation rate was calculated after 1 month.

### 4.17 Metastatic capacity of gastric cancer

MKN45 cells (5 × 10^6^) transduced with lentivrus carrying either zyxin or vector alone were injected in the peritoneal cavity of nude mice. The mice were sacrificed at either day 14 and 28 for evaluation as described previously.^44^

### 4.18 Immunohistochemical staining

Frozen tumor sections were fixed and immersed in sodium citrate buffer to retrieve the antigen. The sections were then treated with anhydrous methanol containing 0.3% H_2_O_2_, and blocked with an immunostaining blocking solution (Beyotime; P0102) for 1 hour at room temperature. After sequential incubation with the primary and secondary antibodies (Gene Tech; GK500705), the sections were incubated in DAB chromogenic solution for 2 min and counterstained with hematoxylin for 5 min.

### 4.19 Co‐immunoprecipitation

The Co‐IP assay was performed using a commercial kit (Absin; abs955). The cells were lysed in lysis buffer containing 1% PMSF. After centrifugation at 14,000×*g* to remove insoluble particles, the supernatant was used as IP sample. After preincubation with Protein A/G agarose beads to remove nonspecific bindings, the samples were incubated with primary antibody overnight at 4°C, followed by incubation with Protein A and G agarose beads. After washing, the samples were analyzed by WB.^41^

### 4.20 GEO analysis

All microarray data were downloaded from the GEO database (http://www.ncbi.nih.gov/geo). Box plots are drawn by boxplot of R.

### 4.21 UALCAN analysis

We examined the zyxin expression level from major clinical features such as gastric cancer stages (stages 1, 2, 3, and 4), patient's gender or age, lymph node stage (N0 1, 2, and 3), and TP53 mutation status through the UALCAN website which provides an extensive and interactive study of bioinformatics applying RNA‐seq and clinical data of 31 malignancies from TCGA (http:// ualcan.path.uab.edu/).

### 4.22 Protein expression and clinical characteristics analysis

The levels of zyxin protein and clinicopathological details and general information of gastric cancer patients were collected. All analyses were performed using the stats package of R.

### 4.23 Analysis of the relationship between the expression of zyxin and the prognosis of gastric cancer patients

The prognostic values of zyxin in gastric cancer were analyzed via TIMER (https://cistrome.shinyapps.io/timer), GEPIA (http://gepia.cancer‐pku.cn/), and UALCAN (https://ualcan.path.uab.edu/) database.

### 4.24 Statistical methods

All experiments were repeated more than three times, and the data were processed by Excel, GraphPad Prism, and SPSS Statistics. The data were expressed as means ± SD, and statistical significance was analyzed by two‐tailed unpaired Student's *t*‐test on two experimental conditions or one‐way ANOVA when repeated measures were compared. WB was used to detect the data of gastric cancer patient tissue samples by nonparametric test Mann–Whitney analysis. *N* = 3 in all experiments except the nude mouse tumor formation experiment, where *N* = 6. In all results, *p* < 0.05 indicates that the data difference is statistically significant.

## AUTHOR CONTRIBUTION

*Conceptualization, design of the study, and writing*: Jin Zhou, Yangxin Li, and Yao‐Hua Song. *Acquisition of data, or analysis and interpretation of data*: Jing Lou, Sha Geng, Wei He, Song‐Bai Liu, Xinghong Shi, Ying Chang, Shiyuan Han, and Panting Qian. *Drafting the paper or revising it critically for important intellectual content*: Jin Zhou, Yangxin Li, Yao‐Hua Song, and Hesham M. Amin. All authors have read and approved the final manuscript.

## CONFLICT OF INTEREST STATEMENT

The authors declare no conflict of interest.

## ETHICS STATEMENT

All animal protocols were approved by the Institutional Laboratory Animal Care and Use Committee of Soochow University (No. SUDA20230228A01).

All patients were provided with informed consent. This study was carried out in accordance with the principles of the Declaration of Helsinki, and the study design was approved by Institutional Ethics Committee of the First Affiliated Hospital of Soochow University (approval number: 2022164).

## Supporting information

Supporting Information

## Data Availability

The data included in this study are available upon request from the corresponding author.
