# Supplementary material for: Zyxin inhibits the epithelial–mesenchymal transition process in gastric cancer by upregulating SIRT1
Source: MedComm (2020). 2023 Sep 3;4(5):e357. doi: 10.1002/mco2.357 (PMC10475219; doi:10.1002/mco2.357)
Supplement: Supplementary file 1 — Supporting Information [file MCO2-4-e357-s001.docx]

**Supplementary materials**

**Zyxin inhibits the epithelial-mesenchymal transition process in gastric cancer by up-regulating SIRT1**

**Running title: Zyxin inhibits the progression of gastric cancer**

Jing Lou^1#^, Sha Geng^1#^, Wei He^1#^, Song-Bai Liu^2#^, Xinghong Shi^1^, Ying Chang^1^, Shiyuan Han^1^, Panting Qian^1^, Hesham M Amin^3^,Yao-Hua Song^1^*, Yangxin Li^4^*, Jin Zhou^5^*.

^1^Cyrus Tang Hematology Center, Collaborative Innovation Center of Hematology, Soochow University, National Clinical Research Center for Hematologic Diseases, the First Affiliated Hospital of Soochow University, Suzhou, P.R. China，State Key Laboratory of Radiation Medicine and Protection, Soochow University, Suzhou, 215123, P.R. China

^2^ Suzhou Key laboratory of medical biotechnology, Suzhou vocational health college, Kehua Road 28, Suzhou, P.R. China 215009

^3^Department of Hematopathology, The University of Texas MD Anderson Cancer Center, 1515 Holcombe Boulevard, Houston, Texas, USA

^4^Institute for Cardiovascular Science and Department of Cardiovascular Surgery, First Affiliated Hospital and Medical College of Soochow University, Collaborative Innovation Center of Hematology, Soochow University, Suzhou, Jiangsu 215123, P. R. China

^5^Department of General Surgery，the First Affiliated Hospital of Soochow University, Suzhou, P. R. China

^#^**These authors contributed equally to this work.**

*Correspondence authors:

Yangxin Li, PhD, FAHA

Institute for Cardiovascular Science and Department of Cardiovascular Surgery,

First Affiliated Hospital of Soochow University, Suzhou, Jiangsu 215123, P. R. China Email: yangxin_li@yahoo.com (YL)

Jin Zhou, M.D., Ph.D.

Department of General Surgery，

The First Affiliated Hospital of Soochow University,

Suzhou, China

Email: 13913506369@163.com

Yao-Hua Song, M.D., Ph.D.

Cyrus Tang Hematology Center, Collaborative Innovation Center of Hematology,

Soochow University

199 Ren Ai Road, Suzhou 215123, China

Email: yaohua_song1@yahoo.com

**Supplemental Figure 1.** Correlation between *zyxin* mRNA expression level and cancer stage of gastric cancer through the GSE27342 dataset in GEO (A) and TCGA (B) database.

**Supplemental Figure 2.** Correlation between zyxin expression level and clinicopathological parameters of gastric cancer through the UALCAN database. (A) Patient’s age. (B) Patient’s gender (male/female) (C) Lymph node stage (N0, 1, 2, and 3). (D) TP53 mutation status.

**Supplemental Figure 3.** Kaplan–Meier survival curves comparing the expression of zyxin in gastric cancer in (A) TIMER, (B) GEPIA, and (C) UALCAN database.

**Supplemental Figure 4.** The effect of zyxin overexpression on ZEB1 expression. (A) Western blot analysis of ZEB1 expression in MKN45-Lv5 and MKN45-Zyxin. (B) Western blot grayscale values were determined using Image J software, and the values were normalized based on GAPDH grayscale values.

**_­_**

**Supplemental Figure 5.** The effect of zyxin overexpression on N87 cell migration and invasion. (A) Transwell assay to analyze the migration and invasion ability of N87-Zyxin and control N87-Lv5 cells, scale: 100 μm. (B) The number of cells successfully passing through the chamber in N87-Zyxin and control N87-Lv5 cells was counted and normalized according to the control N87-Lv5.

**Supplemental Figure 6.** The impact of zyxin overexpression on the proliferation, and sensitivity to drug treatment. (A) CCK8 assay to assess the impact of zyxin overexpression on N87 proliferation ability in vitro. (B) Soft agar colony formation assay. (C-D) The impact of zyxin expression on N87 sensitivity to chemotherapy drug 5-Fu (using a concentration of 4mM) and Etoposide (using a concentration of 80uM).

**Supplemental Table 1:** Clinical characteristics of the gastric cancer patients.

| Characteristics | Variable | Total(N=73) | % |
| --- | --- | --- | --- |
| Age |  |  |  |
|  | ≤55 years | 6 | 8.22 |
|  | 56-65 years | 21 | 28.77 |
|  | 66-75 years | 30 | 41.10 |
|  | ≥76 years | 16 | 21.91 |
| Gender |  |  |  |
|  | Male | 51 | 69.86 |
|  | Female | 22 | 30.14 |
| Stage |  |  |  |
|  | I | 10 | 13.70 |
|  | Ⅱ | 12 | 16.44 |
|  | Ⅲ | 36 | 49.31 |
|  | Ⅳ | 15 | 20.55 |
| T classification |  |  |  |
|  | T1 | 6 | 8.22 |
|  | T2 | 4 | 5.48 |
|  | T3 | 0 | 0 |
|  | T4 | 63 | 86.30 |
| Lymph nodes |  |  |  |
|  | N0 | 26 | 35.62 |
|  | N1 | 7 | 9.59 |
|  | N2 | 16 | 21.91 |
|  | N3 | 24 | 32.88 |
| Distant metastasis |  |  |  |
|  | M0 | 58 | 79.45 |
|  | M1 | 15 | 20.55 |

**Supplemental Table 2:** Sequences of RT-qPCR primers.

| Gene | Forward | Reverse |
| --- | --- | --- |
| *Zyxin* | TTCCACATCGCCTGCTTCACCT | CGCAGGTGTTACACTTCTCCAG |
| *CD44* | CTGCCGCTTTGCAGGTGTA | CATTGTGGGCAAGGTGCTATT |
| *OCT4* | CTTGAATCCCGAATGGAAAGGG | GTGTATATCCCAGGGTGATCCTC |
| *NANOG* | GCGCGGTCTTGGCTCACTGC | GCCTCCCAATCCCAAACAATACGA |
| *E-cadherin* | GCCTCCTGAAAAGAGAGTGGAAG | TGGCAGTGTCTCTCCAAATCCG |
| *Vimentin* | AGGCAAAGCAGGAGTCCACTGA | ATCTGGCGTTCCAGGGACTCAT |
| *SNAI1* | TGCCCTCAAGATGCACATCCGA | GGGACAGGAGAAGGGCTTCTC |
| *SNAI2* | ATCTGCGGCAAGGCGTTTTCCA | GAGCCCTCAGATTTGACCTGTC |
| *SNAI3* | TGCACCTGCAAGATCTGTGGCA | AAGGTTGGAGCGGTCGGCAAAG |
| *ZEB1* | GGCATACACCTACTCAACTACGG | TGGGCGGTGTAGAATCAGAGTC |
| *ZEB2* | AATGCACAGAGTGTGGCAAGGC | CTGCTGATGTGCGAACTGTAGG |
| *GRHL2* | CGCCTATCTCAAAGACGACCAG | CCAGGGTGTACTGAAATGTGCC |
| *OVOL1* | CCTCAAGAGACACGTCCGAACT | GCGTACTTCTGCTGCACACCAT |
| *OVOL2* | CCACAACCAGGTGAAAAGACACC | CGCTGGGTGAAGGCTTTATTGC |
| *SIRT1* | TAGACACGCTGGAACAGGTTGC | CTCCTCGTACAGCTTCACAGTC |
| *GAPDH* | ACCCAGAAGACTGTGGATGG | CAGTGAGCTTCCCGTTCAG |

**Supplemental Table 3:** The CDS sequences of zyxin and the sequences of zyxin overexpression PCR primers.

| Gene | Sequence |
| --- | --- |
| zyxin*-*CDS | ATGGCGGCCCCCCGCCCGTCTCCCGCGATCTCCGTTTCGGTCTCGGCTCCGGCTTTTTACGCCCCGCAGAAGAAGTTCGGCCCTGTGGTGGCCCCAAAGCCCAAAGTGAATCCCTTCCGGCCCGGGGACAGCGAGCCTCCCCCGGCACCCGGGGCCCAGCGCGCACAGATGGGCCGGGTGGGCGAGATTCCCCCGCCGCCCCCGGAAGACTTTCCCCTGCCTCCACCTCCCCTTGCTGGGGATGGCGACGATGCAGAGGGTGCTCTGGGAGGTGCCTTCCCGCCGCCCCCTCCCCCGATCGAGGAATCATTTCCCCCTGCGCCTCTGGAGGAGGAGATCTTCCCTTCCCCGCCGCCTCCTCCGGAGGAGGAGGGAGGGCCTGAGGCCCCCATACCGCCCCCACCACAGCCCAGGGAGAAGGTGAGCAGTATTGATTTGGAGATCGACTCTCTGTCCTCACTGCTGGATGACATGACCAAGAATGATCCTTTCAAAGCCCGGGTGTCATCTGGATATGTGCCCCCACCAGTGGCCACTCCATTCAGTTCCAAGTCCAGTACCAAGCCTGCAGCCGGGGGCACAGCACCCCTGCCTCCTTGGAAGTCCCCTTCCAGCTCCCAGCCTCTGCCCCAGGTTCCGGCTCCGGCTCAGAGCCAGACACAGTTCCATGTTCAGCCCCAGCCCCAGCCCAAGCCTCAGGTCCAACTCCATGTCCAGTCCCAGACCCAGCCTGTGTCTTTGGCTAACACCCAGCCCCGAGGGCCCCCAGCCTCATCTCCGGCTCCAGCCCCTAAGTTTTCTCCAGTGACTCCTAAGTTTACTCCTGTGGCTTCCAAGTTCAGTCCTGGAGCCCCAGGTGGATCTGGGTCACAACCAAATCAAAAATTGGGGCACCCCGAAGCTCTTTCTGCTGGCACAGGCTCCCCTCAACCTCCCAGCTTCACCTATGCCCAGCAGAGGGAGAAGCCCCGAGTGCAGGAGAAGCAGCACCCCGTGCCCCCACCGGCTCAGAACCAAAACCAGGTGCGCTCCCCTGGGGCCCCAGGGCCCCTGACTCTGAAGGAGGTGGAGGAGCTGGAGCAGCTGACCCAGCAGCTAATGCAGGACATGGAGCATCCTCAGAGGCAGAATGTGGCTGTCAACGAACTCTGCGGCCGATGCCATCAACCCCTGGCCCGGGCGCAGCCAGCCGTCCGCGCTCTAGGGCAGCTGTTCCACATCGCCTGCTTCACCTGCCACCAGTGTGCGCAGCAGCTCCAGGGCCAGCAGTTCTACAGTCTGGAGGGGGCGCCGTACTGCGAGGGCTGTTACACTGACACCCTGGAGAAGTGTAACACCTGCGGGGAGCCCATCACTGACCGCATGCTGAGGGCCACGGGCAAGGCCTATCACCCGCACTGCTTCACCTGTGTGGTCTGCGCCCGCCCCCTGGAGGGCACCTCCTTCATCGTGGACCAGGCCAACCGGCCCCACTGTGTCCCCGACTACCACAAGCAGTACGCCCCGAGGTGCTCCGTCTGCTCTGAGCCCATCATGCCTGAGCCTGGCCGAGATGAGACTGTGCGAGTGGTCGCCCTGGACAAGAACTTCCACATGAAGTGTTACAAGTGTGAGGACTGCGGGAAGCCCCTGTCGATTGAGGCAGATGACAATGGCTGCTTCCCCCTGGACGGTCACGTGCTCTGTCGGAAGTGCCACACTGCTAGAGCCCAGACCTGA |
| Overexpression-zyxin-F | CATGCATGCATGTACCCATACGACGTCCCAGACTACGCTGCGGCCCCCCGCCCGTCTC |
| Overexpression-zyxin-R | CGCGGATCCTCAGGTCTGGGCTCTAGCAGTGTGGC |
